# Supplementary material for: Going Retro, Going Viral: Experiences and Lessons in Drug Discovery from COVID-19
Source: Molecules. 2022 Jun 14;27(12):3815. doi: 10.3390/molecules27123815 (PMC9228142; doi:10.3390/molecules27123815)

Sample loading information for gels shown in Main figures and repeats

Fig 2CD r1-1: repeat 1 for Fig. 2C/D. Left to right: no inhibitor, AT9010 (12.5, 25, 50, 100, 200 μM), STP (12.5, 25, 50, 100, 200 μM), GTP (12.5, 25, 50, 100, 200 μM).

Fig 2CD r1-2: repeat 1 for Fig. 2C/D. Left to right: no inhibitor, 2'N3-GTP (12.5, 25, 50, 100, 200 μM), 2'N3-UTP (12.5, 25, 50, 100, 200 μM), RTP (12.5, 25, 50, 100, 200 μM).

Fig 2CD r2: repeat 2 for Fig. 2C/D. Top gel, left to right: no inhibitor, AT9010 (12.5, 25, 50, 100 μM), STP (12.5, 25, 50, 100 μM), RTP (12.5, 25, 50, 100 μM); Bottom gel, left to right: 2'N3-GTP (12.5, 25, 50, 100 μM), 2'N3-UTP (12.5, 25, 50, 100 μM), GTP (12.5, 25, 50, 100 μM).

Fig 2CD r3: repeat 3 for Fig. 2C/D. Top gel, left to right: no inhibitor, AT9010 (12.5, 25, 50, 100 μM), STP (12.5, 25, 50, 100 μM), RTP (12.5, 25, 50, 100 μM); Bottom gel, left to right: 2'N3-GTP (12.5, 25, 50, 100 μM), 2'N3-UTP (12.5, 25, 50, 100 μM), GTP (12.5, 25, 50, 100 μM).

Fig 3B r1: Fig. 3B repeat 1. Left to right: no inhibitor, 1% DMSO, AT-DP, AT-TP, SDP, STP, AT-DP, AT-TP, SDP, STP, AT-DP, AT-TP, SDP, STP.

Fig 3B r2: Fig. 3B repeat 2. Left to right: no inhibitor, TOB, RUT, empty lane, no inhibitor, TOB, RUT, empty lane, TOB, RUT.

Fig 4C: the gel shown in Fig. 4C. All relevant lanes are labeled; numbers after inhibitor names correspond to μM. No inhibitor control = DMSO. To make the figure, the panels were rearranged to match the order of inhibitors in other panels. In addition, the panels with another control inhibitor were removed.

Fig 4D r1: Fig. 4D repeat 1. Lane 1 to 10: No RdRp, 1% DMSO, RFB (100, 300 μM), FDX (50, 150 μM), RPN (100, 300 μM), SUR (10, 30 μM).

Fig 4D r2: Fig. 4D repeat 2. Lane 1 to 10: No RdRp, 1% DMSO, RFB (100, 300 μM), FDX (50, 150 μM), RPN (100, 300 μM), SUR (10, 30 μM).

Fig 4D r3: Fig. 4D repeat 3. Lane 1 to 10: No RdRp, 1% DMSO, RFB (100, 300 μM), FDX (50, 150 μM), RPN (100, 300 μM), SUR (10, 30 μM).

Fig 4E r1: Fig. 4E repeat 1. Lane 1 to 9: 1% DMSO, RFB (100, 300 μM), FDX (50, 150 μM), RPN (100, 300 μM), SUR (10, 30 μM).

Lane 10 to 18: 1% DMSO, RFB (100, 300 μM), FDX (50, 150 μM), RPN (100, 300 μM), SUR (10, 30 μM).

Fig 4E r2: Fig. 4E repeat 2. Left to right: 1% DMSO, RFB (100, 300 μM), FDX (50, 150 μM), RPN (100, 300 μM), SUR (10, 30 μM).

Fig 5B: the gel shown in Fig. 5B; Fig. 5B_r2 and _r3 are repeats. All lanes are labeled on images. None = DMSO.

File/Range: F:\Original gels\Fig 2CD r1-1.gel / 0.000-4965 Counts /Magnification - 1.47
User Name: Artsimovitch Lab
Image Name: F:\Original gels\Fig 2CD r1-1.gel
Image Comment: Mode=Phosphorimaging
Method=[Phosphor]
Laser=635nm
Filter=[IP] (ch.1)
PMT Type=Bialkali
PMT=ch.1,800V
Shading=IP_2011-02-28T14_59_40
Inverted=NO
Stage=Phosphor Stage
MainOffset=25mm
SubOffset=25mm

Present Date/Time: 2022:06:01 17:07:50

Fig. 2CD r1-1

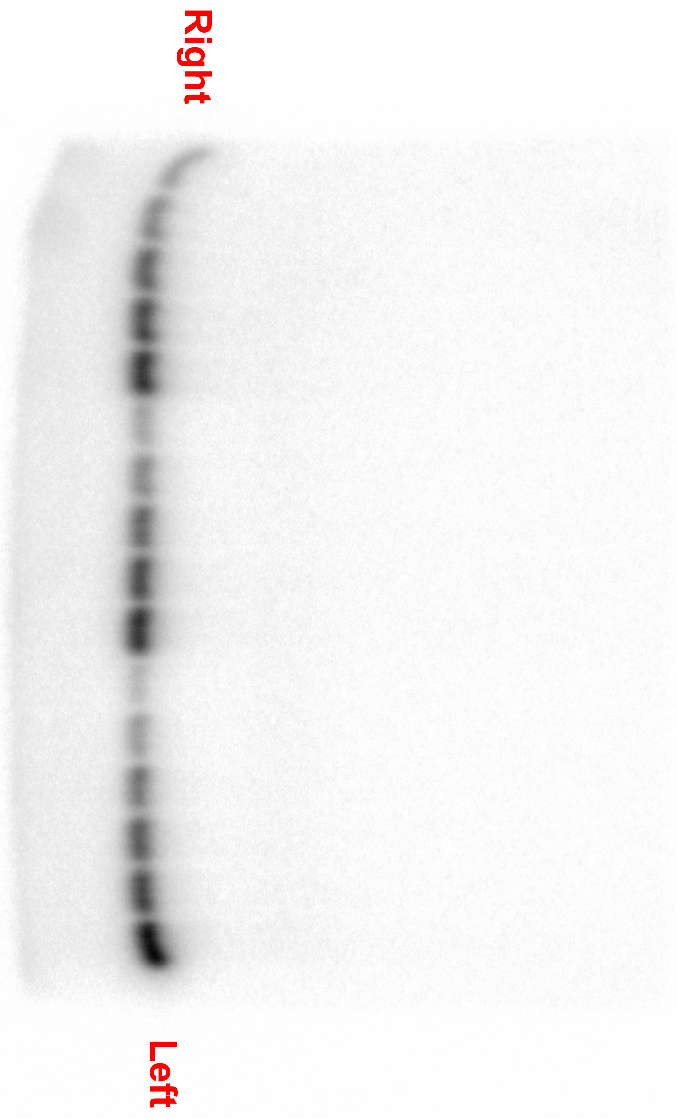

File/Range: F:\Original gels\Fig 2CD r1-2.gel / 0.000-7492 Counts /Magnification - 1.47
User Name: Artsimovitch Lab
Image Name: F:\Original gels\Fig 2CD r1-2.gel
Image Comment: Mode=Phosphorimaging
Method=[Phosphor]
Laser=635nm
Filter=[IP] (ch.1)
PMT Type=Bialkali
PMT=ch.1,800V
Shading=IP_2011-02-28T14_59_40
Inverted=NO
Stage=Phosphor Stage
MainOffset=25mm
SubOffset=25mm

Present Date/Time: 2022:06:01 17:08:39

Fig. 2CD r1-2

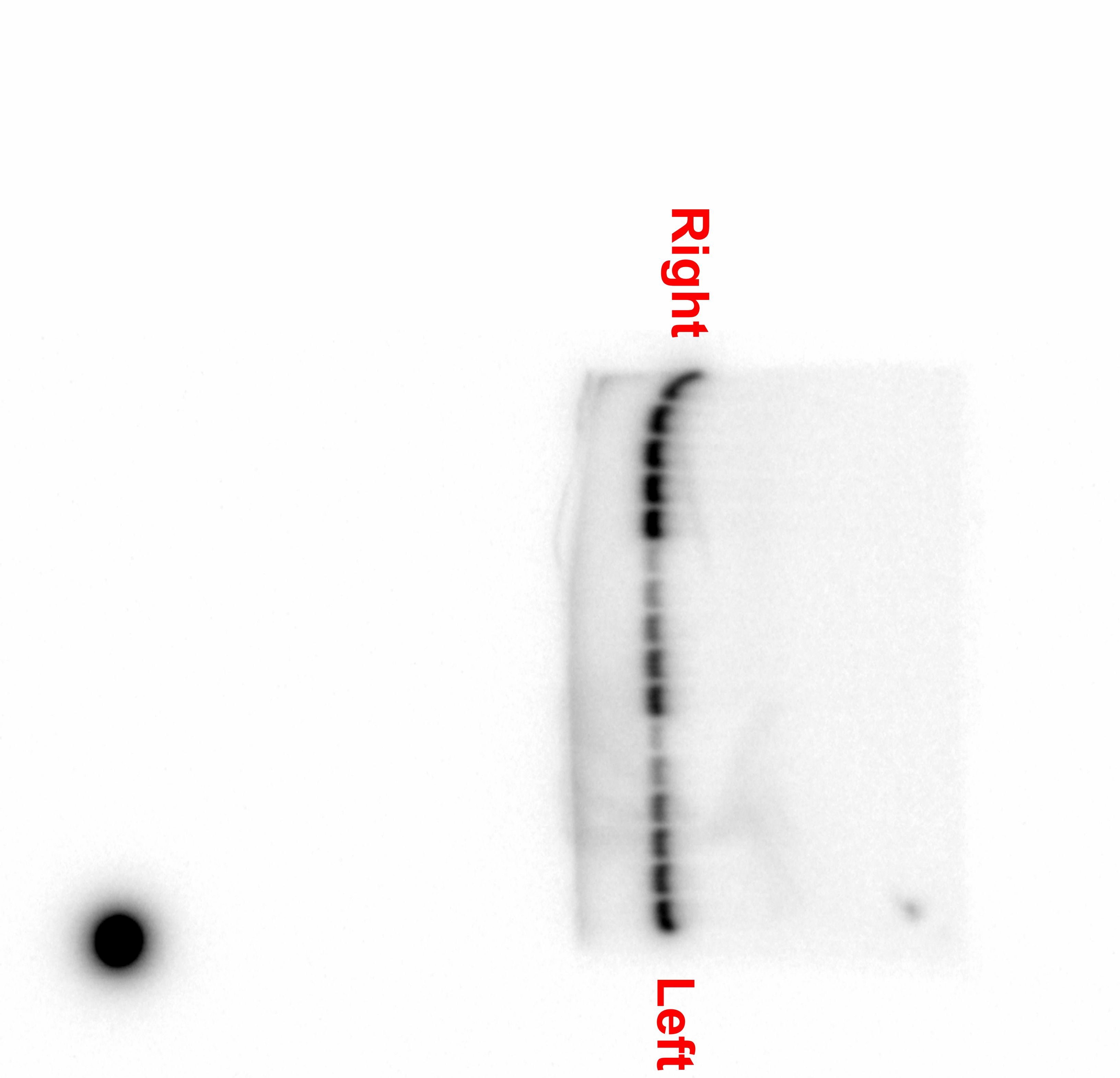

File/Range: F:\Original gels\Fig 2CD r2.gel / 0.000-3742 Counts /Magnification - 1.00
User Name: Artsimovitch Lab
Image Name: F:\Original gels\Fig 2CD r2.gel
Image Comment: Mode=Phosphorimaging
Method=[Phosphor]
Laser=635nm
Filter=[IP] (ch.1)
PMT Type=Bialkali
PMT=ch.1,800V
Shading=IP_2011-02-28T14_59_40
Inverted=NO
Stage=Phosphor Stage
MainOffset=25mm
SubOffset=25mm

Present Date/Time: 2022:06:01 17:16:28
Scan Date/Time: 2022:03:16 14:51:27
Prep. Date/Time: 2022:03:16 14:51:27

Fig. 2CD r3

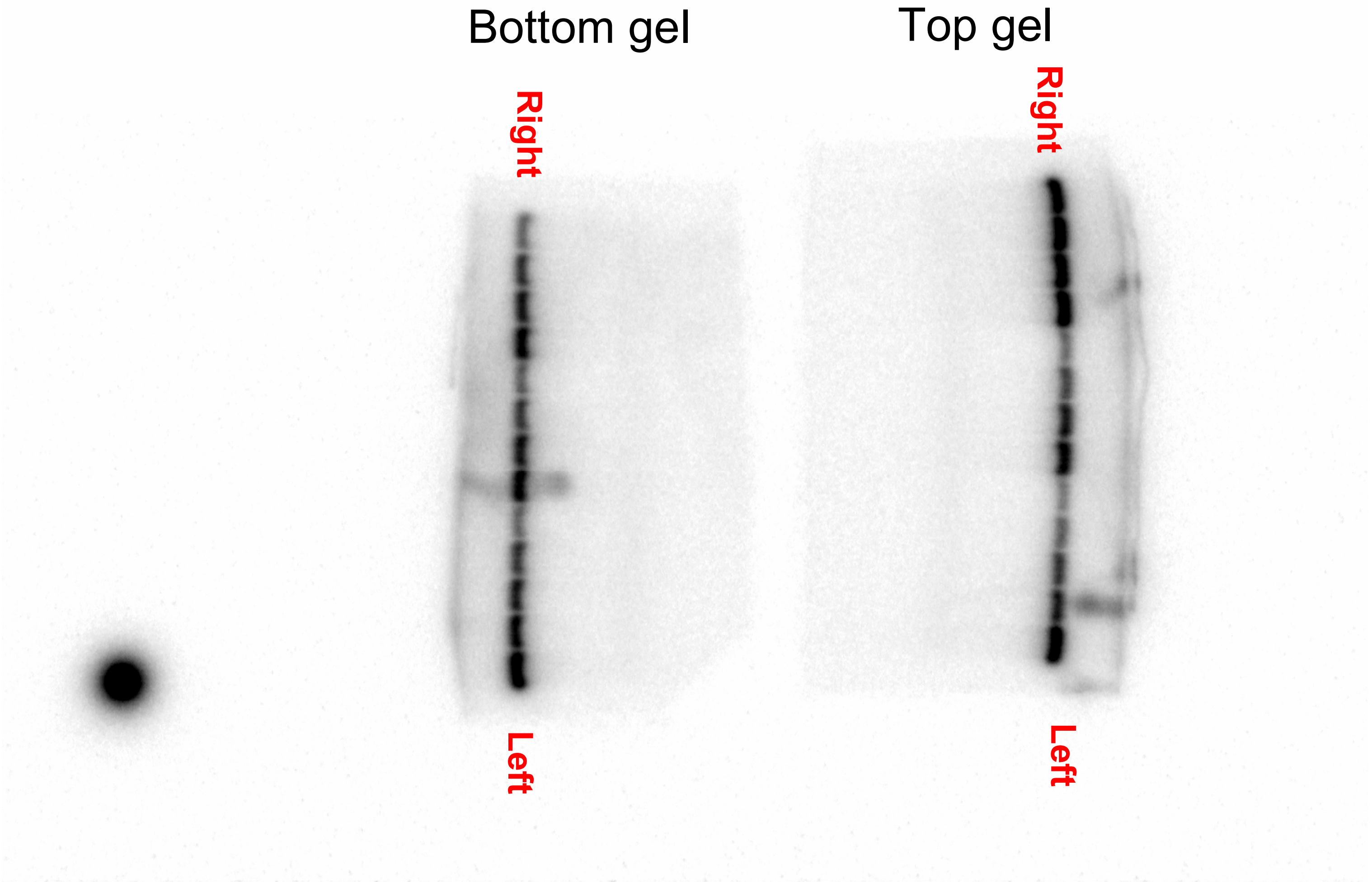

File/Range: F:\Original gels\Fig 2CD r3.gel / 0.000-1997 Counts /Magnification - 1.00
User Name: Artsimovitch Lab
Image Name: F:\Original gels\Fig 2CD r3.gel
Image Comment: Mode=Phosphorimaging
Method=[Phosphor]
Laser=635nm
Filter=[IP] (ch.1)
PMT Type=Bialkali
PMT=ch.1,800V
Shading=IP_2011-02-28T14_59_40
Inverted=NO
Stage=Phosphor Stage
MainOffset=25mm
SubOffset=25mm

Present Date/Time: 2022:06:01 17:16:54
Scan Date/Time: 2022:03:16 16:07:30
Prep. Date/Time: 2022:03:16 16:07:30

Fig. 2CD r4

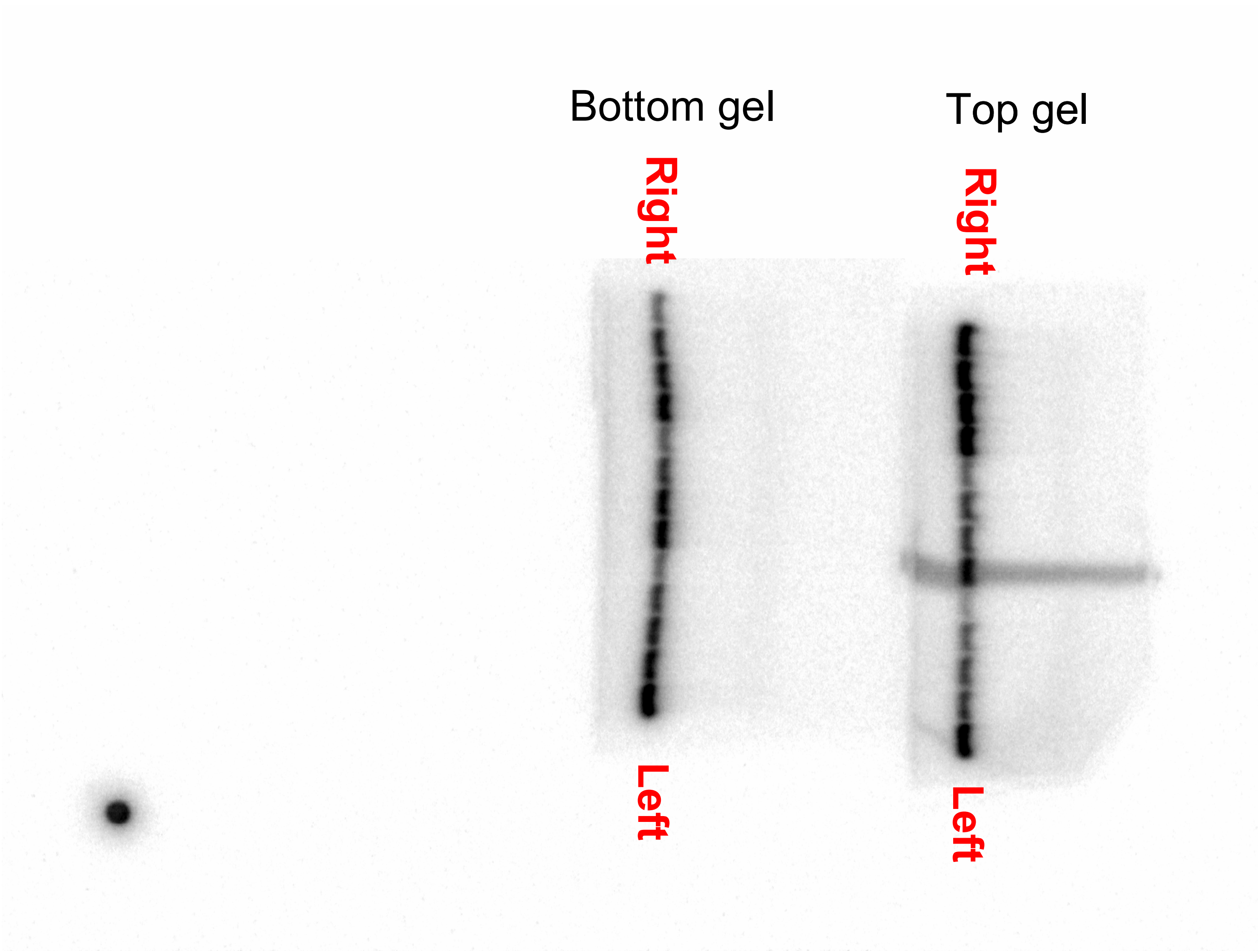

File/Range: F:\Original gels\Fig 3B r1.gel / 0.000-357.6 Counts /Magnification - 1.47
User Name: Artsimovitch Lab
Image Name: F:\Original gels\Fig 3B r1.gel
Image Comment: Mode=Phosphorimaging
Method=[Phosphor]
Laser=635nm
Filter=[IP] (ch.1)
PMT Type=Bialkali
PMT=ch.1,650V
Shading=IP_2011-02-28T14_59_40
Inverted=NO
Stage=Phosphor Stage
MainOffset=25mm
SubOffset=25mm

Fig. 3B r1

Present Date/Time: 2022:06:01 17:09:46

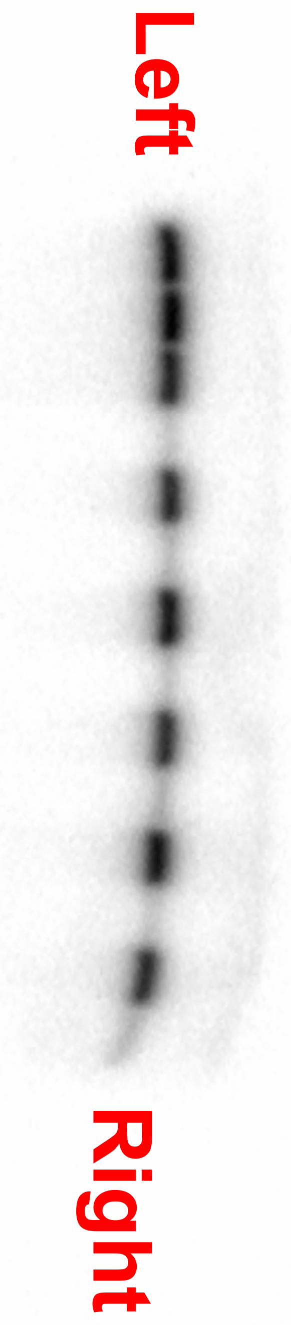

File/Range: F:\Original gels\Fig 3B r2.gel / 0.000-11950 Counts /Magnification - 1.47
User Name: Artsimovitch Lab
Image Name: F:\Original gels\Fig 3B r2.gel
Image Comment: Mode=Phosphorimaging
Method=[Phosphor]
Laser=635nm
Filter=[IP] (ch.1)
PMT Type=Bialkali
PMT=ch.1,650V
Shading=IP_2011-02-28T14_59_40
Inverted=NO
Stage=Phosphor Stage
MainOffset=25mm
SubOffset=25mm

Present Date/Time: 2022:06:01 17:10:03

Fig. 3B r2

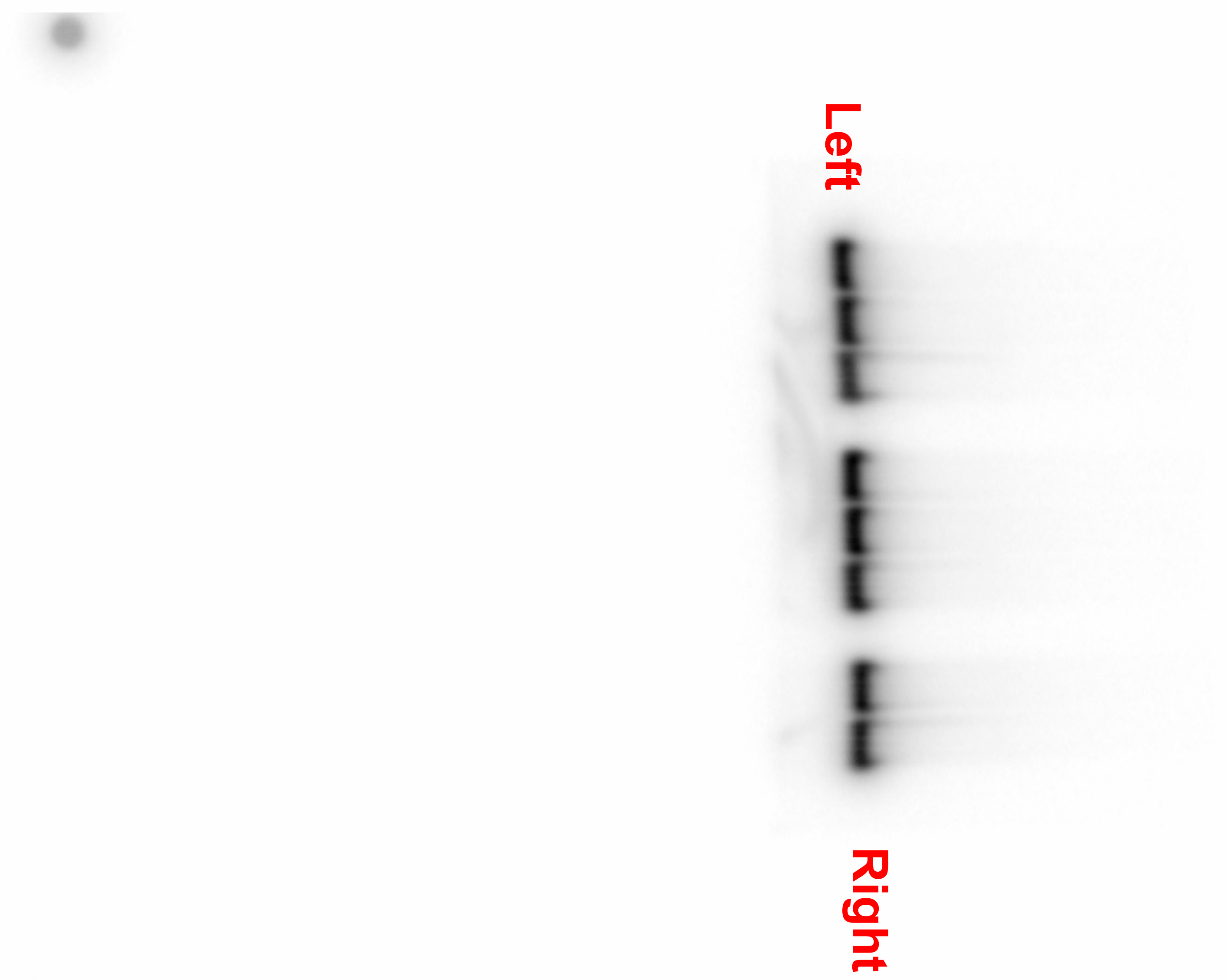

File/Range: F:\Original gels\Fig 4C.gel / 48.08-5616 Counts /Magnification - 0.50
User Name: Artsimovitch Lab
Image Name: F:\Original gels\Fig 4C.gel
Image Comment: Mode=Fluorescence
Method=[Cy5]
Laser=635nm
Filter=[LPR] (ch.1)
PMT Type=Bialkali
PMT=ch.1,900V
Shading=635-Ch1_2011-02-28T09_24_36
Inverted=NO
Stage=Fluorescence Stage
MainOffset=0mm
SubOffset=50mm

Present Date/Time: 2022:06:01 17:19:23
Scan Date/Time: 2022:03:16 15:33:28
Prep. Date/Time: 2022:03:16 15:33:28

Fig. 4C

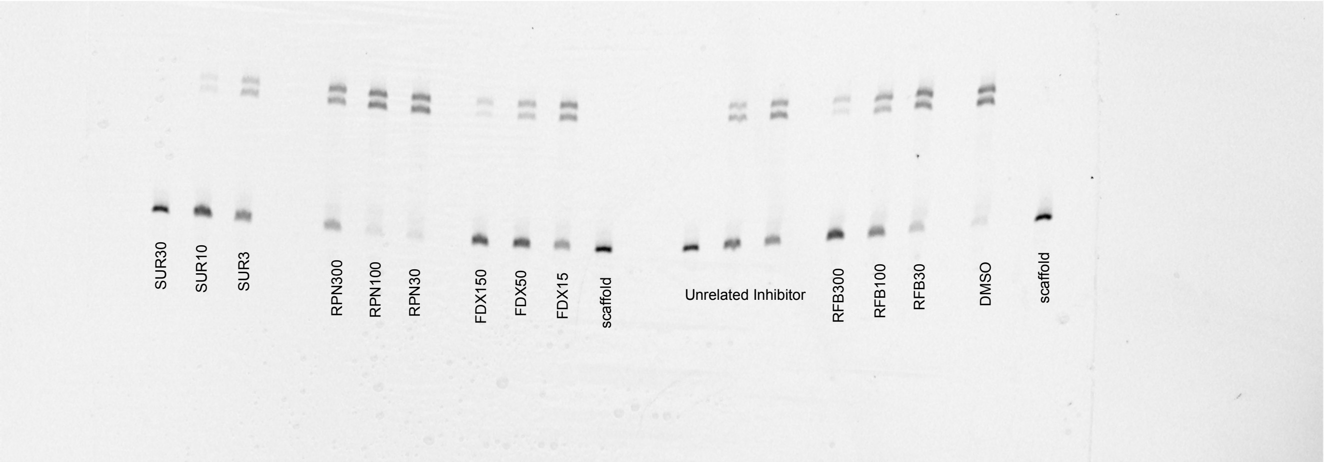

File/Range: F:\Original gels\Fig 4D r1.gel / 14.64-10002 Counts /Magnification - 1.00
User Name: Artsimovitch Lab
Image Name: F:\Original gels\Fig 4D r1.gel
Image Comment: Mode=Fluorescence
Method=[Cy5]
Laser=635nm
Filter=[LPR] (ch.1)
PMT Type=Bialkali
PMT=ch.1,800V
Shading=635-Ch1_2011-02-28T09_24_36
Inverted=NO
Stage=Fluorescence Stage
MainOffset=25mm
SubOffset=25mm

Present Date/Time: 2022:06:01 17:20:55
Scan Date/Time: 2022:03:21 13:09:49
Prep. Date/Time: 2022:03:21 13:09:49

Fig. 4D r1

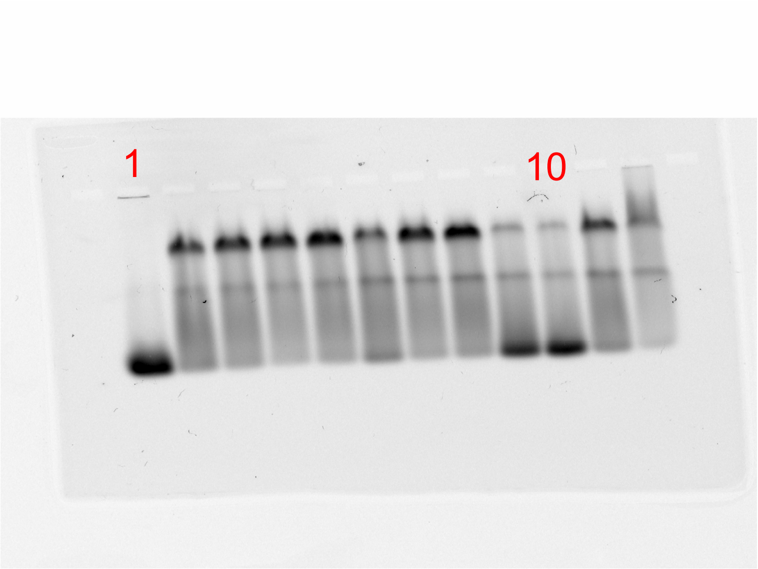

File/Range: F:\Original gels\Fig 4D r2.gel / 58.20-5190 Counts /Magnification - 1.00
User Name: Artsimovitch Lab
Image Name: F:\Original gels\Fig 4D r2.gel
Image Comment: Mode=Fluorescence
Method=[Cy5]
Laser=635nm
Filter=[LPR] (ch.1)
PMT Type=Bialkali
PMT=ch.1,800V
Shading=635-Ch1_2011-02-28T09_24_36
Inverted=NO
Stage=Fluorescence Stage
MainOffset=25mm
SubOffset=25mm

Present Date/Time: 2022:06:01 17:21:21
Scan Date/Time: 2022:03:22 12:43:28
Prep. Date/Time: 2022:03:22 12:43:28

Fig. 4D r2

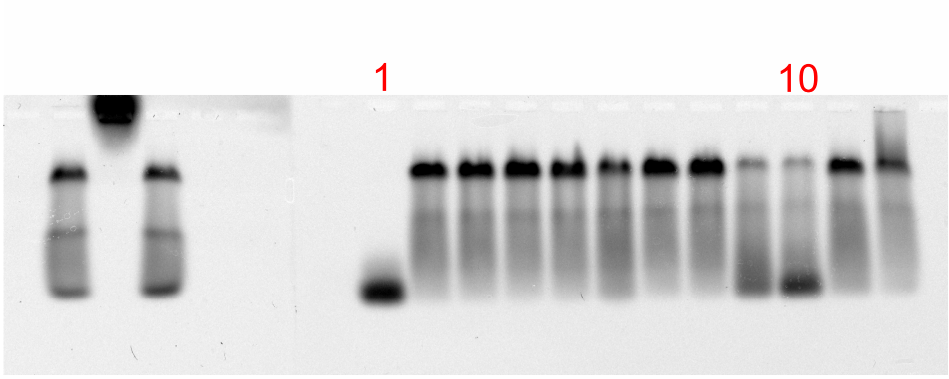

File/Range: F:\Original gels\Fig 4D r3.gel / 134.8-22797 Counts /Magnification - 2.21
User Name: Artsimovitch Lab
Image Name: F:\Original gels\Fig 4D r3.gel
Image Comment: Mode=Fluorescence
Method=[Cy5]
Laser=635nm
Filter=[LPR] (ch.1)
PMT Type=Bialkali
PMT=ch.1,900V
Shading=635-Ch1_2011-02-28T09_24_36
Inverted=NO
Stage=Fluorescence Stage
MainOffset=25mm
SubOffset=25mm

Present Date/Time: 2022:06:01 17:11:19
Scan Date/Time
Prep. Date/Time

Fig. 4D r3

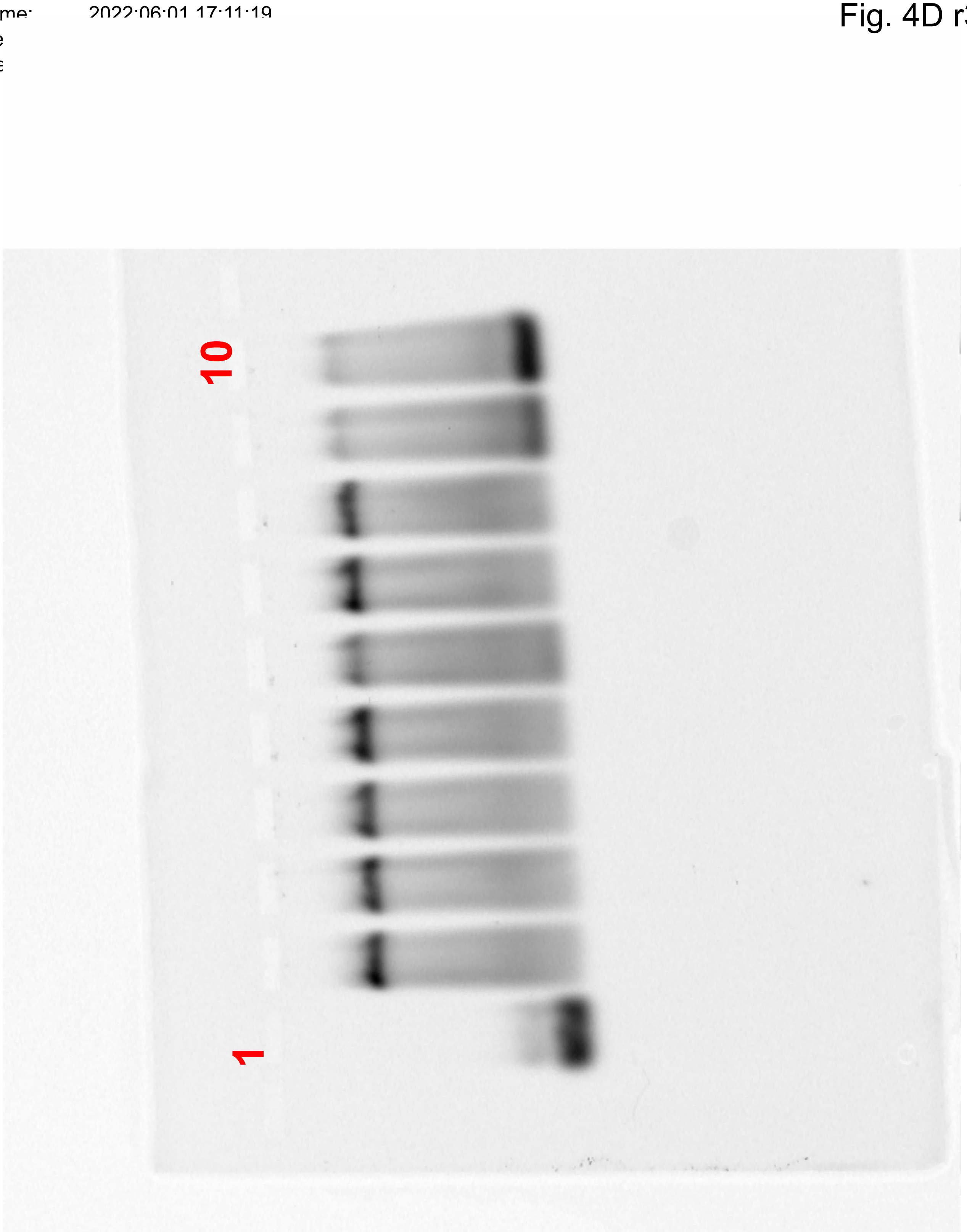

File/Range: F:\Original gels\Fig 4E r1.gel / 0.201-1270 Counts /Magnification - 1.00
User Name: Artsimovitch Lab
Image Name: F:\Original gels\Fig 4E r1.gel
Image Comment: Mode=Phosphorimaging
Method=[Phosphor]
Laser=635nm
Filter=[IP] (ch.1)
PMT Type=Bialkali
PMT=ch.1,800V
Shading=IP_2011-02-28T14_59_40
Inverted=NO
Stage=Phosphor Stage
MainOffset=25mm
SubOffset=25mm

Present Date/Time: 2022:06:01 17:21:47
Scan Date/Time: 2022:03:22 12:54:30
Prep. Date/Time: 2022:03:22 12:54:30

Fig. 4E r1

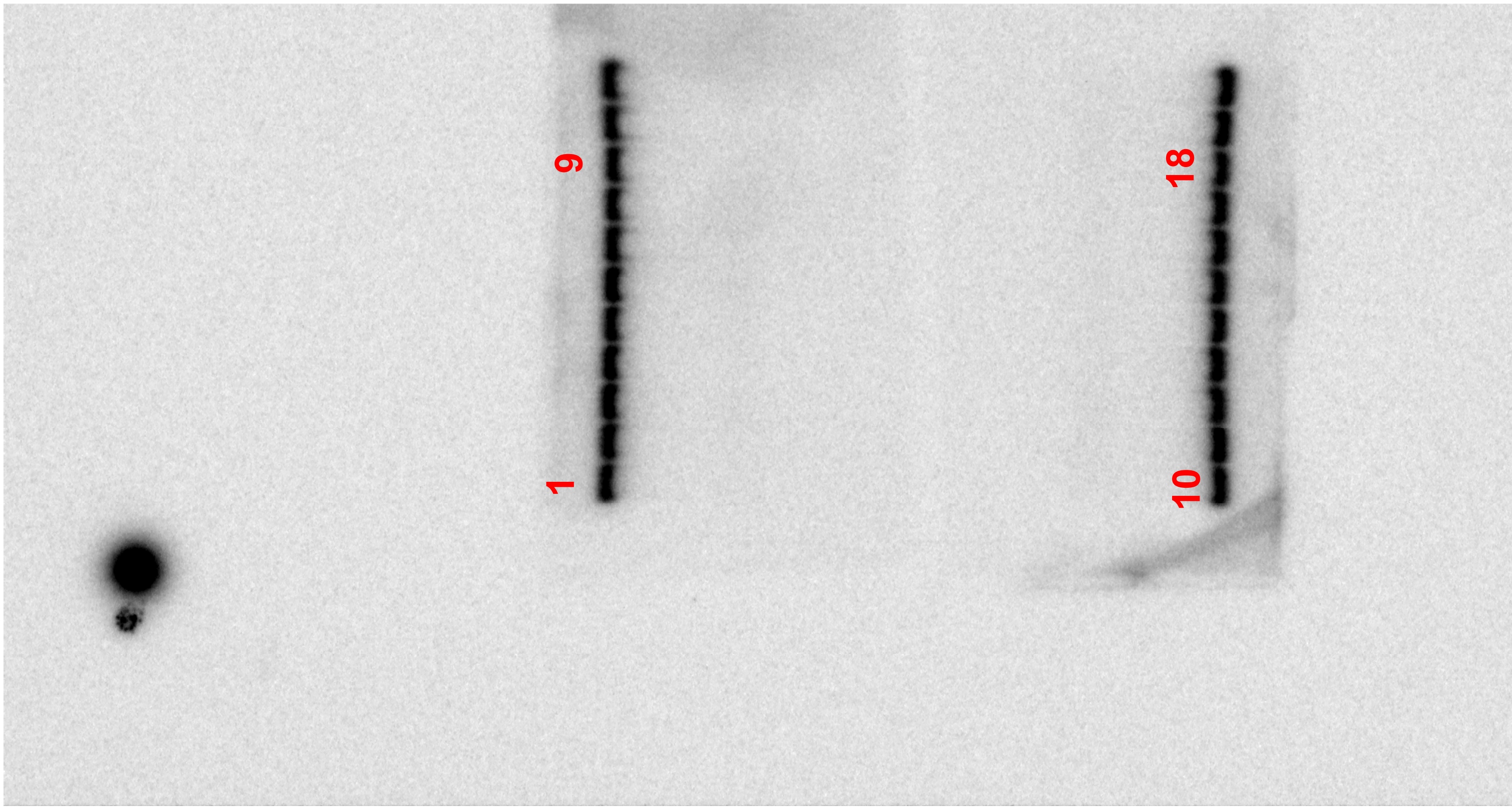

File/Range: F:\Original gels\Fig 4E r2.gel / 0.000-4853 Counts /Magnification - 1.11
User Name: Artsimovitch Lab
Image Name: F:\Original gels\Fig 4E r2.gel
Image Comment: Mode=Phosphorimaging
Method=[Phosphor]
Laser=635nm
Filter=[IP] (ch.1)
PMT Type=Bialkali
PMT=ch.1,650V
Shading=IP_2011-02-28T14_59_40
Inverted=NO
Stage=Phosphor Stage
MainOffset=25mm
SubOffset=25mm

Present Date/Time: 2022:06:01 17:11:57

Fig. 4E r2

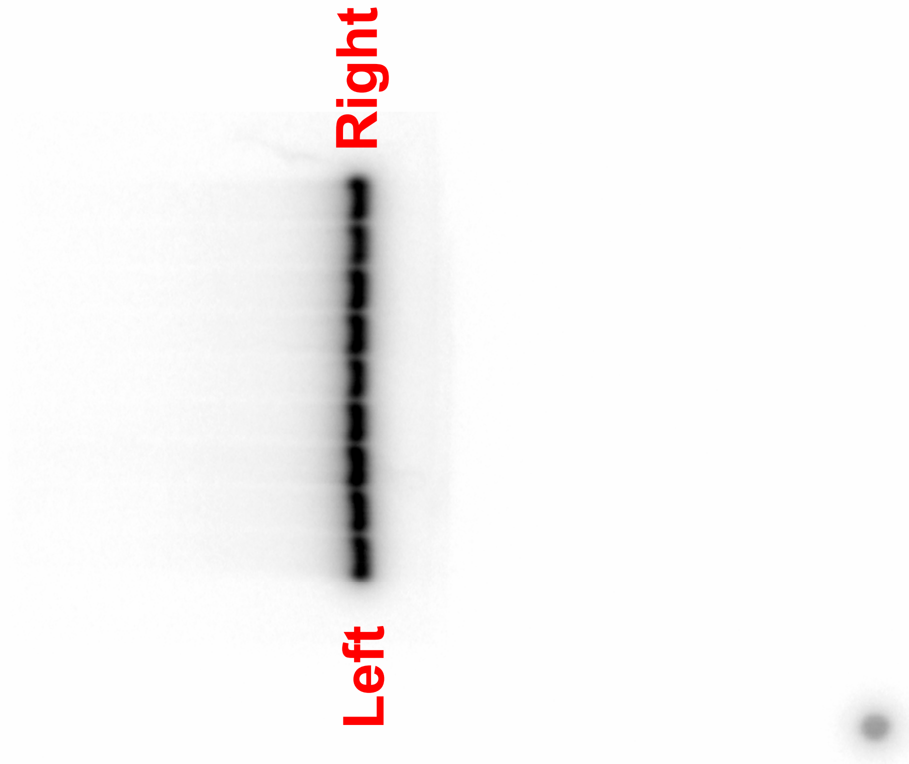

File/Range: F:\Original gels\Fig 5B.gel / 128.3-2801 Counts /Magnification - 0.50
User Name: Artsimovitch Lab
Image Name: F:\Original gels\Fig 5B.gel
Image Comment: Mode=Fluorescence
Method=[Cy5]
Laser=635nm
Filter=[LPR] (ch.1)
PMT Type=Bialkali
PMT=ch.1,1000V
Shading=635-Ch1_2011-02-28T09_24_36
Inverted=NO
Stage=Fluorescence Stage
MainOffset=0mm
SubOffset=25mm

Present Date/Time: 2022:06:01 17:23:17
Scan Date/Time: 2022:03:18 13:24:34
Prep. Date/Time: 2022:03:18 13:24:34

Fig. 5B

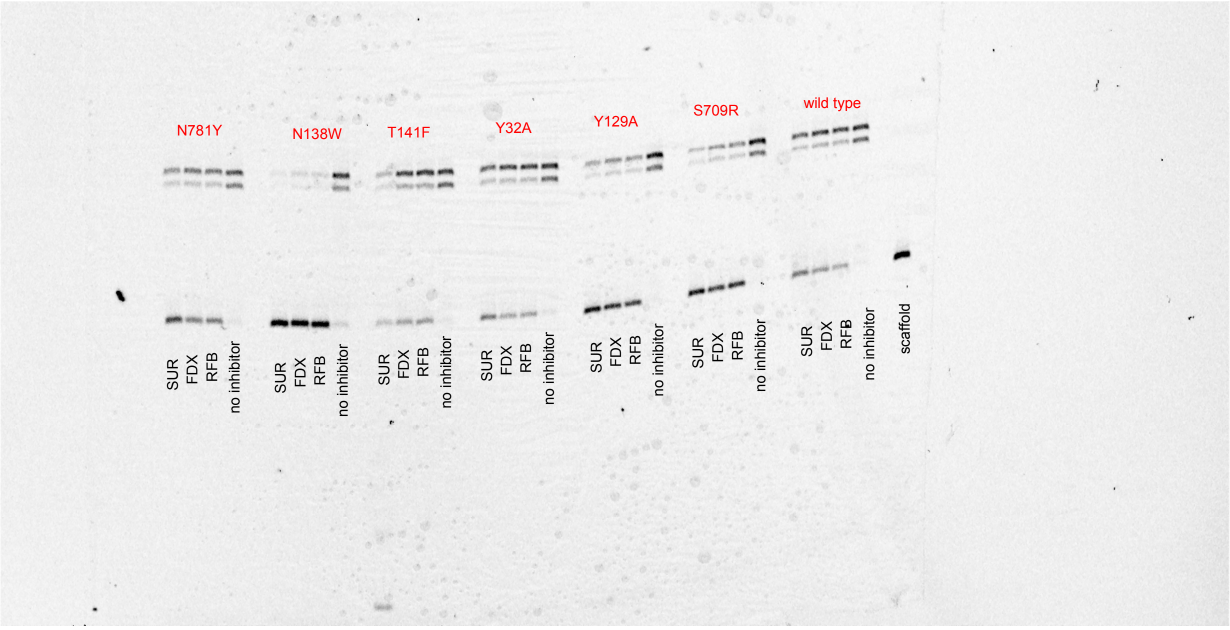

File/Range: F:\Original gels\Fig 5B_R2.gel / 39.23-5001 Counts /Magnification - 0.50
User Name: Artsimovitch Lab
Image Name: F:\Original gels\Fig 5B_R2.gel
Image Comment: Mode=Fluorescence
Method=[Cy5]
Laser=635nm
Filter=[LPR] (ch.1)
PMT Type=Bialkali
PMT=ch.1,900V
Shading=635-Ch1_2011-02-28T09_24_36
Inverted=NO
Stage=Fluorescence Stage
MainOffset=0mm
SubOffset=0mm

Present Date/Time: 2022:06:01 17:23:42
Scan Date/Time: 2022:03:17 11:29:30
Prep. Date/Time: 2022:03:17 11:29:30

Fig. 5B r1

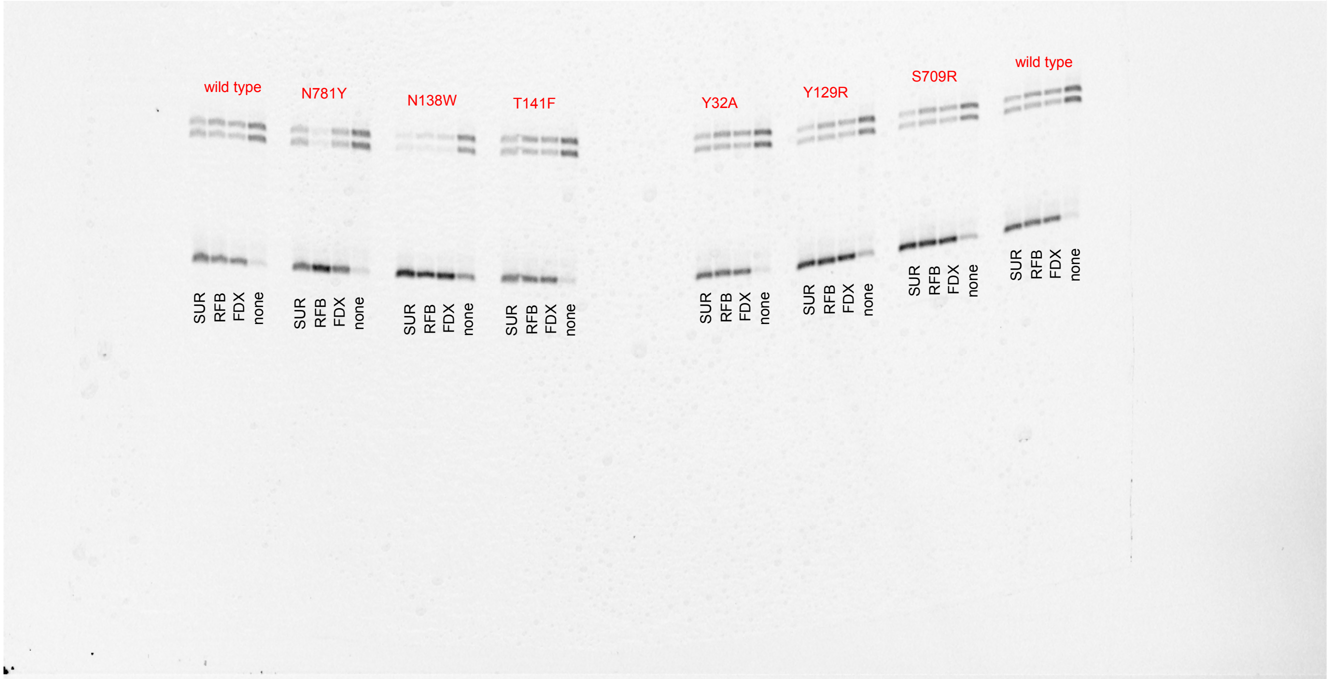

File/Range: F:\Original gels\Fig 5B_R3.gel / 122.2-2404 Counts /Magnification - 0.50
User Name: Artsimovitch Lab
Image Name: F:\Original gels\Fig 5B_R3.gel
Image Comment: Mode=Fluorescence
Method=[Cy5]
Laser=635nm
Filter=[LPR] (ch.1)
PMT Type=Bialkali
PMT=ch.1,900V
Shading=635-Ch1_2011-02-28T09_24_36
Inverted=NO
Stage=Fluorescence Stage
MainOffset=0mm
SubOffset=25mm

Present Date/Time: 2022:06:01 17:23:59
Scan Date/Time: 2022:03:17 16:27:15
Prep. Date/Time: 2022:03:17 16:27:15

Fig. 5B r2

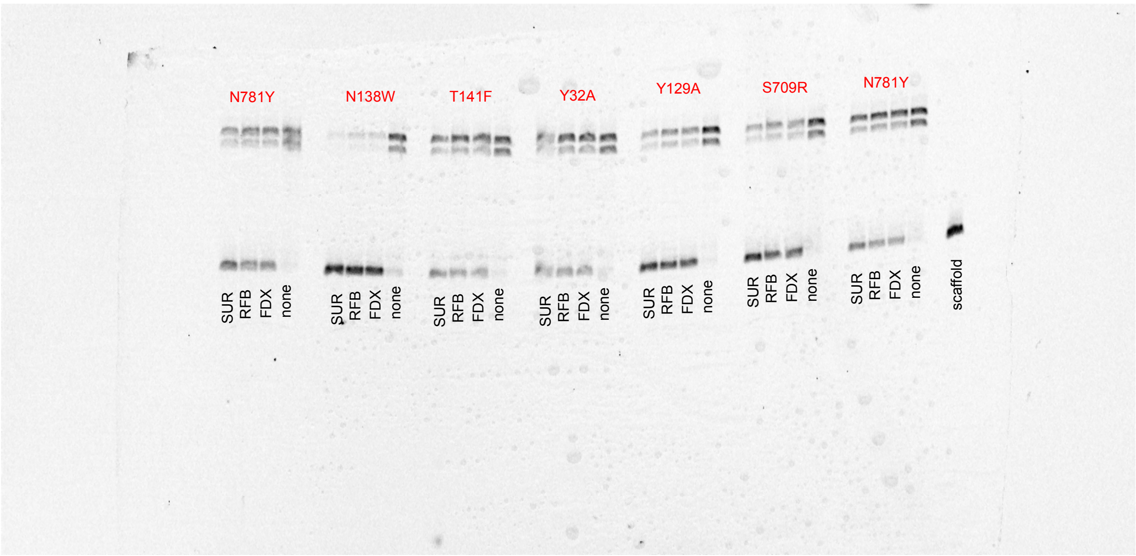

Supplement: Supplementary file 1 [file molecules-27-03815-s001.zip › Gel_files.pdf]
