# Supplementary material for: Going Retro, Going Viral: Experiences and Lessons in Drug Discovery from COVID-19
Source: Molecules. 2022 Jun 14;27(12):3815. doi: 10.3390/molecules27123815 (PMC9228142; doi:10.3390/molecules27123815)
Supplement: Supplementary file 1 [file molecules-27-03815-s001.zip › Table S1.pdf]

Table S1. Plasmids

Name	Key features/sequence	Source; Addgene#
pIA1362	T7 promoter–His_8-GB1-TEV-nsp7	[25]; 166860
pIA1363	T7 promoter–His_8-GB1-TEV-nsp8	[25]; 166861
pIA1414	T7 promoter–His_{10}-SUMO-nsp9	[23]; 172402
pIA1400	T7 promoter–His_{10}-SUMO-nsp12	[23]; 172519
pIA1401	T7 promoter–His_{10}-SUMO-nsp12[S709R]	This work
pIA1402	T7 promoter–His_{10}-SUMO-nsp12[Y129A]	[25]; 172403
pIA1403	T7 promoter–His_{10}-SUMO-nsp12[Y32A]	This work
pIA1407	T7 promoter–His_{10}-SUMO-nsp12[T141F]	This work
pIA1411	T7 promoter–His_{10}-SUMO-nsp12[N138W]	This work
pIA1417	T7 promoter–His_{10}-SUMO-nsp12[N781Y]	This work
